# The frequency of cooking dinner at home and its association with nutrient intake adequacy among married young-to-middle-aged Japanese women: the POTATO Study

**DOI:** 10.1017/jns.2019.9

**Published:** 2019-04-22

**Authors:** Aki Saito, Mai Matsumoto, Aiko Hyakutake, Masafumi Saito, Naoko Okamoto, Masayoshi Tsuji

**Affiliations:** 1 Department of Nutritional Epidemiology and Shokuiku, National Institutes of Biomedical Innovation, Health and Nutrition, Tokyo, Japan; 2 Department of Nutrition and Food Science, Graduate School of Humanities and Sciences, Ochanomizu University, Tokyo, Japan; 3 Department of Human Nutrition, Seitoku University, Chiba, Japan; 4 Department of Nutrition, Faculty of Nutrition, Kobe Gakuin University, Hyogo, Japan; 5 Department of Clinical Dietetics and Human Nutrition, Faculty of Pharmaceutical Sciences, Josai University, Saitama, Japan; 6 Department of Health and Nutrition, Osaka Shoin Women's University, Osaka, Japan; 7 Department of Preventive Medicine and Public Health, Fukuoka University, Fukuoka, Japan

**Keywords:** Women, Cooking, Dietary intake, Japan, Nutrient adequacy, BDHQ, brief-type self-administered diet history questionnaire, DG, tentative dietary goal for preventing lifestyle-related diseases, DRI, dietary reference intake, EAR, estimated average requirement

## Abstract

Home cooking has been suggested as a key to healthy dietary intakes. However, little is known about the association between cooking behaviour and nutrient intake among young-to-middle-aged women. We aimed to investigate the association between home cooking frequency and nutrient intake adequacy among married Japanese women. Self-administered questionnaires were used to assess the weekly frequency of cooking dinner at home and habitual nutrient intake during the preceding month. We evaluated nutrient intake adequacy by comparing the self-reported intake with two indices of the dietary reference intakes for Japanese (2015): the estimated average requirement (EAR) of fourteen nutrients, and the ‘tentative dietary goal for preventing lifestyle-related diseases’ (DG) of seven nutrients. A total of 143 participants (25–44 years old) completed the questionnaires, with 32·9 % of participants reporting a weekly home cooking frequency of seven times/week. Women with a higher home cooking frequency (seven times/week) were more likely to have children (*P* = 0·001) than those with a lower home cooking frequency (0–6 times/week). Of the nutrients evaluated, there was no significant difference between the two groups in meeting EAR and DG. Our findings suggest that daily home cooking may not be necessary to achieve adequate nutrient intake, specifically among married, young-to-middle-aged Japanese women.

Poor nutritional quality and the negative health consequences that come with it are associated with a high frequency of eating food prepared away from home^(^^1^^,^^2^^)^. Conversely, high frequency of food preparation at home is associated with longer life expectancy^(^^3^^)^, and prevention of weight gain and type 2 diabetes^(^^4^^)^. Lower frequency of cooking at home is thought to lead to increased consumption of away-from-home food or eating out, and the food consumed in this manner is usually high in fat and Na^(^^5^^)^. Those who frequently cook at home are likely to have better nutritional knowledge and cooking skills than those who do not, which can improve nutritional intake^(^^6^^–^^8^^)^.

Home cooking behaviours are influenced by various factors, and not solely by nutritional knowledge or cooking skills. A systematic review of observational studies showed a complex set of factors influencing home cooking behaviour, including sex, personal relationships, time availability, employment, ethnicity and culture^(^^9^^)^. Thus, determinants of home cooking behaviour are important factors ultimately influencing health status, and are critical to consider in establishing effective dietary interventions among various cultural populations.

In previous studies dietary quality reportedly improved with home meal preparation among elementary school children and adolescents^(^^10^^,^^11^^)^, adults aged approximately 20 years^(^^8^^)^ and middle-aged women^(^^12^^)^. Life stage may have a direct impact on home cooking behaviour. In a previous study, home cooking frequency differed by age, particularly for women, with less time spent cooking in the younger generation (45 years or younger) and more time spent cooking among women aged 45 years or older^(^^13^^)^. One possible explanation is that the younger generation is subject to more time constraints due to responsibilities such as earning a living and taking care of family. A transition to parenthood is more likely at this age than for other age groups, which could influence both dietary intake^(^^14^^)^ and time spent cooking^(^^13^^)^. However, the study was limited by excluding analysis of meal preparation frequency and individual cooking behaviours. Another study suggests that mothers and wives have a perceived role to provide nutritious meals for the household^(^^15^^)^, which may also have an impact on frequency of cooking at home and dietary quality. Living with a partner is associated with a higher home food preparation frequency, according to a nationwide study conducted in the USA^(^^16^^)^. These studies suggest that women of reproductive age in Western countries may play important roles in establishing the nutritional status of household members, yet it is not known whether the roles are similar for Asian women. In Japan, the gender gap in married couples is wider than in Western countries^(^^17^^)^, and married women spend significantly more time on household chores, including cooking, than married men (4·6 *v.* 0·5 h/d)^(^^18^^)^. Besides, over 90 % of Japanese married couples live away from their parents^(^^19^^)^, which may lead to the household work being a bigger burden for a woman in the household. However, it has not been well studied whether home cooking frequency affects dietary intake among married, young-to-middle-aged women in Asian countries.

In this study, we examined whether the frequency of home cooking is associated with improved dietary intake in married, young-to-middle-aged, Japanese women. We hypothesised that greater home cooking frequency is associated with better nutritional intake within this population.

## Methods

### Procedure

This study examined the data from participants who responded to a cross-sectional, self-administered questionnaire survey (the POTATO study). The details of the study protocol have been described elsewhere^(^^20^^)^. In short, between June and December 2014 and between July and August 2015, married women aged 23–44 years who lived in Japan were recruited through volunteer study collaborators (recruiters) via online advertisements using social media, such as Facebook, or through word of mouth. A recruiter explained the survey's purpose and outlined the procedure to 353 married women who were interested in participating. These married women were then asked to complete questionnaires about dietary habits and lifestyle. A total of 241 women completed both questionnaires (response rate = 68·3 %). This study was conducted according to the guidelines laid down in the Declaration of Helsinki, and all procedures involving human subjects were approved by the Ethics Committee of the Seitoku University (no. H25U017). Written informed consent was obtained from all participants.

### Study population

Among those who reported their cooking frequency (*n* 212), the present analyses were limited to participants who reported that they ate dinner/supper every day (*n* 205). We excluded the following respondents: those who were receiving nutritional counselling (*n* 2), those who were pregnant or lactating (*n* 56), those who did not report the number of family members (*n* 3), and those who reported energy intake less than half the energy requirement for the lowest physical-activity category according to the dietary reference intakes (DRI) for Japanese (2015)^(^^21^^)^, or those reporting equal to or more than 1·5 times the energy requirement for the highest physical activity category (*n* 2). The final sample thus consisted of 143 Japanese married women aged 25–44 years, after excluding sixty-two respondents (one duplicate).

### Home cooking frequency

Home cooking frequency was assessed in the lifestyle questionnaire, using the following question: ‘During the past 1 month, how many times per week did you cook dinner at home for your family and for yourself? This includes working in the kitchen for more than 10 min, and also includes helping someone to cook. Do not include preparing tables or washing dishes’. Possible answers ranged from 0 to 7 dinners cooked at home per week.

### Dietary assessment

Dietary habits during the preceding month were assessed using a previously validated brief-type self-administered diet history questionnaire (BDHQ)^(^^22^^,^^23^^)^. The BDHQ is a structured questionnaire that includes questions about intake frequencies of selected foods commonly consumed in Japan, general dietary behaviours and common cooking methods. The daily intake estimates for foods (fifty-eight items in total), energy and selected nutrients were calculated using an *ad hoc* computer algorithm developed for the BDHQ, based on the Standard Tables of Food Composition in Japan^(^^24^^)^. Validity of the BDHQ and its structure, as well as that of the methods used to calculate dietary intake, have been explained in detail in previous studies^(^^22^^,^^23^^)^. Briefly, in a study of ninety-two women aged 31–69 years, the median Pearson's correlation coefficients of nutrient intake between the BDHQ and 16-d weighed dietary records was 0·54 (range 0·34–0·87)^(^^22^^)^. Although dietary supplement use was queried in the lifestyle questionnaire, intake via supplements was not included in the analysis because of the lack of a reliable composition table for dietary supplements in Japan.

Reported dietary intakes were adjusted using estimated energy requirement (EER) to minimise errors inherent to self-reporting before comparing the reported nutrient intakes with the Japanese DRI values^(^^21^^)^ as described in previous studies^(^^25^^,^^26^^)^. The value of EER was calculated for age and according to the Japanese DRI for modest physical activity. Adjustment was made using the following equation: energy-adjusted intake (units/d) = observed intake (units/d) × EER (kJ/d)/observed energy intake (kJ/d). Intake of fat, saturated fat, carbohydrate and protein was calculated as a percentage of daily energy intake using reported values (crude).

### Determination of nutritional intake adequacy

Adequacy of intake for each nutrient was determined by comparing the adjusted level for each nutrient with its corresponding Japanese DRI value using a previously reported method^(^^25^^–^^27^^)^. In the Japanese DRI, multiple indices are established as reference ranges with different purposes. The estimated average requirement (EAR) is set to determine sufficiency of nutrient intake, and the ‘tentative dietary goal for preventing lifestyle-related diseases’ (DG) is set as an intake goal to prevent non-communicable, lifestyle-related disease. An intake level below the EAR was designated as ‘not meeting EAR’ using the cut-point method for thirteen nutrients, which included protein, vitamin A (expressed as retinol activity equivalents), thiamine, riboflavin, niacin (expressed as niacin equivalents), vitamin B_6_, vitamin B_12_, folate, vitamin C, Ca, Mg, Zn and Cu. Due to the strongly skewed distribution of Fe requirements among menstruating women^(^^28^^,^^29^^)^, a probability method was used instead of the cut-point method. Fe intake of <9·3 mg/d (probability of inadequacy >50 % for menstruating women whose bioavailability of Fe is 15 %)^(^^30^^)^ was considered as inadequate. An intake level outside the DG range was considered as not meeting the standard for the seven nutrients studied, which included protein, total fat, saturated fat, carbohydrate, total dietary fibre, Na (expressed as salt-equivalents) and K. Although the EAR is set for biotin, Cr, Mo, Se and iodine in the Japanese DRI, these nutrients were excluded from the present study because food composition tables in Japan are lacking.

### Assessment of lifestyle variables

In the lifestyle questionnaire, the participants reported on their educational background (junior high school, high school, junior college or vocational technical school, or university), working status (full-time, part-time, or other), household income (less than 2 million yen/year, 2 million to 6 million yen/year, 6 million to 10 million yen/year, or more than 10 million yen/year), current smoking status (yes or no), the use of dietary supplements (yes or no), and whether they had children aged younger than 18 years (yes or no). Data regarding self-reported body heights (cm) and weights (kg) were also obtained from the BDHQ. BMI was calculated as the body weight (kg) divided by the square of the body height (m^2^). Based on the reported home address, participants were grouped into one of six residential blocks (Hokkaido and Tohoku, Kanto, Hokuriku and Tokai, Kinki, Chugoku and Shikoku, or Kyushu), and into three categories according to population size of the geographical area of residence (city with a population of ≥1 million, city with a population of <1 million, and town or village).

### Statistical analysis

Because 32·9 % of the participants reported their weekly home cooking frequency as seven, the participants were categorised into two groups: those who cooked meals seven times/week and those cooking less frequently (0–6 times/week). Basic characteristics in these two groups were compared using independent-samples *t* tests for continuous variables, and the χ^2^ test for categorical variables. The categories of educational background and annual household income were regrouped due to the sample sizes of certain categories: *n* 0 for junior high school, and *n* 1 for less than 2 000 000 yen/year.

The means and standard deviations of energy and nutrient intake were calculated for each group, and significant differences across the groups were assessed using independent-samples *t* tests. The percentage of participants whose intake were below the EAR or outside the range of the DG was calculated. Logistic regression analysis was used to examine the difference in the prevalence of participants ‘not meeting DRI’ between the groups, adjusting for potential confounding factors. To examine the overall nutritional adequacy of each participant's dietary intake, we counted the number of nutrients that did not meet each EAR and DG. The difference between groups in the number of nutrients ‘not meeting DRI’ was assessed using ANCOVA. Potential confounding factors considered in the analyses were those indicating differences between the two groups (*P* < 0·1) and were reportedly associated with dietary intake^(^^20^^,^^31^^)^ (the presence of children aged younger than 18 years in the household and size of residential area), or which could affect the response of the BDHQ, since the questionnaire was designed to assess dietary intake during the preceding month^(^^22^^,^^23^^)^ (the survey year). All statistical analyses were performed using SAS statistical software, version 9.4 (SAS Institute Inc.). All reported *P* values were two-tailed, and a *P* value <0·05 was considered as statistically significant.

## Results

Basic characteristics of the participants are shown in Table 1. The mean age of the subjects was 32·4 (sd 4·5) years. The number of participants who reported a home cooking frequency of seven times/week was forty-seven (32·9 %), six times was thirty-seven (25·9 %), five times was twenty-five (17·5 %), four times was twelve (8·4 %), three times was eight (5·6 %), two times was nine (6·3 %), one time was two (1·4 %) and no times was three (2·1 %). Compared with participants with a lower (0–6 times/week) home cooking frequency, those with a higher home cooking frequency (seven times/d) were more likely to have children aged younger than 18 years in the household, and to have had participated in the study in 2014 (*P* < 0·001 and *P* = 0·023, respectively). No significant differences were observed in age, BMI, distribution of educational backgrounds, working status, annual household income, current smoking status, supplement use, residential blocks, and the population size of residential areas between the two groups.

**Table 1.** Demographics of study participants according to weekly frequency of cooking dinner at home (Mean values and standard deviations; numbers and percentages)

			Weekly frequency of cooking dinner at home				
	Total (n 143)		7 times/week (n 47)		0–6 times/week (n 96)		
	*n*	%	*n*	%	*n*	%	P*
Age (years)							0·324
Mean	32·4		33·0		32·2		
sd	4·5		4·6		4·4		
BMI (kg/m^2^)							0·585
Mean	20·3		20·2		20·4		
sd	2·5		2·3		2·6		
Having children aged younger than 18 years							<0·001
No	82	57·3	16	34·0	66	68·8	
Yes	61	42·7	31	66·0	30	31·3	
Educational background							0·602
University or higher	106	74·1	34	72·3	72	75·0	
Junior college or vocational technical school	28	19·6	11	23·4	17	17·7	
High school	9	6·3	2	4·3	7	7·3	
Working status							0·602
Full-time worker	70	49·0	18	38·3	52	54·2	
Part-time worker	22	15·4	6	12·8	16	16·7	
Others	51	35·7	23	48·9	28	29·2	
Annual household income							0·602
More than 10 000 000 yen	22	15·4	5	10·6	17	17·7	
6 000 000 yen to 10 000 000 yen	63	44·1	21	44·7	42	43·8	
Less than 6 000 000 yen	58	40·6	21	44·7	37	38·5	
Current smoking							0·388
No	137	95·8	46	97·9	91	94·8	
Yes	6	4·2	1	2·1	5	5·2	
Dietary supplement user							0·939
No	101	70·6	33	70·2	68	70·8	
Yes	42	29·4	14	29·8	28	29·2	
Residential block							0·340
Hokkaido and Tohoku	11	7·7	2	4·3	9	9·4	
Kanto	74	51·8	21	44·7	53	55·2	
Hokuriku and Tokai	16	11·2	6	12·8	10	10·4	
Kinki	17	11·9	6	12·8	11	11·5	
Chugoku and Shikoku	12	8·4	7	14·9	5	5·2	
Kyusyu	13	9·1	5	10·6	8	8·3	
Population size of residential area							0·080
City with a population >1 million	52	36·4	13	27·7	39	40·6	
City with a population <1 million	79	55·2	27	57·5	52	54·2	
Town or village	12	8·4	7	14·9	5	5·2	
Survey year							0·023
2014	108	75·5	41	87·2	67	69·8	
2015	35	24·5	6	12·8	29	30·2	

* Means for continuous values were compared between the groups using an independent-samples *t* test, and proportions for categorical values were compared using the χ^2^ test.

Table 2 shows the energy and nutrient intakes according to home cooking frequency. There was no significant difference in the intake status (not meeting DRI) of any of the nutrients between study groups, after adjusting for the presence of children in the household, residential area size, and the survey year.

**Table 2.** Daily nutrient intakes and prevalence of not meeting dietary reference intakes (DRI) (tentative dietary goals for preventing lifestyle-related disease (DG) and estimated average requirements (EAR)) among 143 married women according to weekly frequency of cooking dinner at home*†

				Weekly frequency of cooking dinner at home						
				7 times/week (*n* 47)			0–6 times/week (*n* 96)			
			Reference value	Mean	sd	Not-meeting DRI (%)‡	Mean	sd	Not-meeting DRI (%)‡	*P*§
Energy	kJ/d	–		7372	2146	–	6761	1402	–	–
Fat	% Energy	DG	20–30	27·7	4·3	36·2	28·5	4·7	41·7	0·671
Saturated fat	% Energy	DG	≤7	7·6	1·6	61·7	8·0	1·8	69·8	0·636
Protein	% Energy	DG	13–20	15·6	3·0	12·8	15·0	2·1	21·9	0·122
Carbohydrate	% Energy	DG	50–65	53·2	6·6	29·8	52·6	6·0	32·3	0·621
Dietary fibre	g/d	DG	≥18	14·0	3·1	83·0	13·6	3·9	86·5	0·585
Na (salt-equivalent)||	g/d	DG	<7	11·0	2·0	100·0	10·9	2·0	97·9	0·933
K	mg/d	DG	≥2600	2907	649	31·9	2754	699	50·0	0·083
Protein	g/d	EAR	≥40	77·1	14·7	0·0	74·0	10·3	0·0	–
Vitamin A¶	μg RE/d	EAR	≥450	852	369	10·6	896	381	7·3	0·444
Thiamine	mg/d	EAR	≥0·9	0·9	0·2	55·3	0·9	0·2	66·7	0·195
Riboflavin	mg/d	EAR	≥1	1·4	0·3	4·3	1·4	0·3	10·4	0·134
Niacin**	mg NE/d	EAR	≥9	32·1	7·8	0·0	30·4	6·0	0·0	–
Vitamin B_6_	mg/d	EAR	≥1	1·5	0·3	4·3	1·4	0·3	11·5	0·201
Vitamin B_12_	μg/d	EAR	≥2	10·4	5·9	0·0	8·6	4·1	0·0	–
Folate	μg/d	EAR	≥200	386	116	2·1	384	140	3·1	0·636
Vitamin C	mg/d	EAR	≥85	125	42	17·0	121	51	29·2	0·113
Ca	mg/d	EAR	≥550	619	197	36·2	571	162	45·8	0·331
Mg	mg/d	EAR	≥230	281	52	21·3	266	56	36·5	0·226
Fe††	mg/d	EAR	≥8·5	8·8	1·9	68·1	8·6	2·0	69·8	0·929
Zn	mg/d	EAR	≥6	9·1	1·1	2·1	8·8	1·0	0·0	0·945
Cu	mg/d	EAR	≥0·6	1·3	0·2	0·0	1·2	0·2	0·0	–

RE, retinol equivalents; NE, niacin equivalents.

* Adjustment of reporting error was performed according to the following equation: energy-adjusted intake (units/d) = observed intake (units/d) × estimated energy requirement (kJ/d)/observed energy intake (kJ/d). For fat, saturated fat, carbohydrate and protein, a percentage from daily energy intake was calculated using reported values (crude).

† Nutrient intakes were not significantly different across the groups (*P* > 0·05).

‡ Percentage of participants whose intake was outside the range of DG or below the EAR. Each energy-adjusted nutrient intake was compared with each DRI value, using the cut-point method.

§ The prevalence of not meeting DRI was compared using logistic regression analysis adjusting for having children or not, population size of residential area, and survey year (2014 or 2015).

|| Na × 2·54.

¶ Sum of retinol, β-carotene/12, α-carotene/24, and cryptoxanthin/24.

** Sum of niacin and protein/6000.

†† The probability of inadequacy of >50 % for menstruating women whose bioavailability of Fe is 15 % (<9·3 mg/d), was considered as not meeting EAR

For overall nutrient intake adequacy, the mean numbers of nutrients with ‘not meeting EAR’ designations were 2·2 (sd 2·0) and 2·8 (sd 2·2) in the participants with a high- and a low-home cooking frequency, respectively (Table 3). The difference was not significant among the two groups after adjusting for confounding factors (*P* = 0·202). The mean numbers of nutrients falling in the ‘not meeting DG’ were 3·6 (sd 1·4) and 4·0 (sd 1·3) in the high- and low-home cooking frequency groups, respectively. No significant difference was observed across the home cooking frequency values between the study groups after adjusting for confounding factors (*P* = 0·178). We replicated our analyses using a different definition of cooking frequency (six times/week or less) and obtained consistent results (data not shown).

**Table 3.** Number of nutrients not meeting dietary reference intakes (tentative dietary goals for preventing lifestyle-related disease (DG) and estimated average requirements (EAR)) status among participants according to the weekly frequency of cooking dinner at home* (Mean values with their standard errors)

	Weekly frequency of cooking dinner at home				
	7 times/week (*n* 47)		0–6 times/week (*n* 96)		
	Mean	se	Mean	se	*P*†
Not meeting EAR	2·5	0·4	3·1	0·3	0·184
Not meeting DG	3·8	0·2	4·1	0·2	0·178

* Adjustment was performed for having children or not, population size of residential area, and survey year (2014 or 2015).

† Significance of each value was compared using ANCOVA.

## Discussion

In this study, we examined the association between home cooking frequency and nutrient intake adequacy in married, young Japanese women. Our results showed that nutrient intake adequacy did not significantly differ according to the home cooking frequency within this population. The nutrients that did not meet Japanese DRI were the same for both groups, with saturated fat and Na intake above the DG and dietary fibre intake below the DG.

Cooking behaviour can be influenced by various factors. In the present study, married women with a high home cooking frequency were more likely to have children. Previous studies have shown that sharing more meals with family members was associated with a higher participation in food preparation by adolescents^(^^8^^)^, and increased home cooking was also linked with having children at home^(^^16^^,^^32^^)^. Such an observation might be because mothers may perceive a responsibility to provide nutritious meals for their children. Moreover, in the present study, even adjusting for the presence of children, the results showed no significant difference in the nutrient intake adequacy according to home cooking frequency. A previous study has reported that among Japanese young women, eating out frequently was associated with inadequate nutrient intake in those living alone, but not in those living with family members^(^^26^^)^. This observation may partly explain the present results because all of our participants lived with their families, which may have mitigated the influence of away-from-home food on the nutritional intake adequacy, especially among those with a lower cooking frequency.

Although the present results showed no association between home cooking frequency and nutrient intake adequacy, several previous studies have suggested various benefits for home-cooked meals^(^^33^^–^^36^^)^. For example, a recent study has reported that consuming daily home-cooked dinners was associated with reduced intake of added sugar, solid fat and energy-dense foods among low-income households in the USA^(^^35^^)^. One study showed that a high frequency of consuming home-cooked dinners (6–7 times/week) was associated with lower intake of fat and sugar than a low frequency of consuming home-cooked dinners (0–1 times/week)^(^^33^^)^. Another study has reported that compared with eating home-cooked dinners less frequently, eating home-cooked dinners more than three times/week was associated with a greater intake of fruits and vegetables, as well as with higher plasma levels of vitamin C^(^^36^^)^. Moreover, more frequent intake of away-from-home meals was reportedly associated with lower serum levels of the vitamins D, E, C, B_6_ and B_12_, folate and carotenoids^(^^34^^)^. These previous studies examined home-cooked meals as meals prepared by the participants or by family members of the participants. The present study only examined the participants’ involvement in cooking dinner, which could explain the difference in our results compared with the above-mentioned studies. Several other studies have limited the analysis to the participants’ own cooking practice and have reported the relationship between home cooking frequency and dietary intake among adult women. Smith *et al*.^(^^37^^)^ have reported that women who shared meal preparation with other household members had higher intakes of vegetables and dairy products than those who did not, although these differences were small. In that study, women who had sole responsibility for meal preparation did not have better dietary intake than other women, and this observation is consistent with our results. In the present study, we could not obtain the frequency of home-cooked meal intake. According to the National Health and Nutrition Survey in Japan, approximately 40 % of young women aged 20–39 years reported that they ate out or used take-away foods more than two times/week^(^^31^^)^. Hence, some of our participants might have eaten out rather than consume a home-cooked meal when they did not prepare a meal at home. Also, we did not obtain the information about which family member is responsible for cooking and who lived in the household besides their husband and children. Further investigation on the influence of home-cooked meals and home cooking behaviour on dietary intake is needed.

Another possible reason for the non-significant relationship between study parameters in this study could be that most of the participants in this study were likely to cook frequently, with more than 95 % of the participants reporting a cooking frequency of three times or more per week. Additionally, we divided the participants into two groups (daily cooks and non-daily cooks); it is possible that the lower frequency of cooking was not low enough to cause a difference in the nutrient intake adequacy in this study. Moreover, the median cooking frequency was six times/week in our participants. The participants in the present study were more likely to cook than those in previous studies, and this could partly explain our results. Thus, to determine any difference in the intake of nutrients, large studies should be designed to ensure a larger number of participants with low cooking frequency (<6 times/week). A previous study of participants with few instances of eating out has suggested that the dietary choices made when eating out were different from the choices made when eating at home, and less healthy dietary choices such as those including sweet and savoury bakery products, soft drinks, juices and non-alcoholic beverages were consumed frequently while eating out^(^^38^^)^. In contrast, a US study has indicated that middle-aged women who spent more time on meal preparation at dinner did not always eat healthier meals^(^^12^^)^. In this previous study, more total fibre, Fe and vegetables were consumed by those who spent more than 20 min for preparing dinner than those who spent less time preparing dinner, while more energy and Na were consumed by those who spent more time in preparing a meal. In the present study, since we focused on habitual dietary intake and home cooking frequency, we did not examine the meal preparation time and what the participants ate when they cooked. Future studies should examine the actual meal preparation time and the meal contents to characterise home meal preparation behaviour.

Despite the lack of difference between groups, there were several nutrients that did not meet EAR or DG in Japanese DRI. Among these, Na intake did not reach the DG values in almost all the participants. Nonetheless, high Na intake has been a major subject of public health concern in Japan^(^^39^^)^. Additionally, high saturated fat and low dietary fibre intake was common in both groups. The inadequacy in the intake of these nutrients is considered a risk factor for non-communicable diseases^(^^40^^–^^42^^)^. The present results may implicate that adequate nutrient intake should be encouraged regardless of meal preparation settings.

This is the first study to examine the association between home cooking frequency and nutrient intake adequacy in young, married Japanese women, with and without children. Our findings suggest that home cooking frequency may not influence the quality of women's own dietary intake, although we only examined the frequency of cooking for dinner. However, the dietary behaviour of women in this age (mid-20s to mid-40s) could exert a great influence on the quality of dietary intake in other family members, because women (as mothers) may have more responsibility for meal preparation in the household^(^^16^^,^^37^^,^^43^^)^. It is also important to note that various factors such as socio-economic status or living conditions may be associated with different dietary behaviours, including cooking^(^^16^^,^^44^^)^. Understanding the impacts of cooking behaviours in the long term is important because children may acquire cooking skills from their parents^(^^45^^)^. As the frequency and quality of meals prepared at home by women of a household may have a direct influence on the quality of dietary intake of other household members, future studies of such possibility are needed in various populations.

Our study has several limitations. First, our study sample was not a random sample of Japanese adult women. Hence, our results cannot be applied to other populations. Due to our recruitment strategy (snowball sampling, mainly through word of mouth), 74·1 % of participants were university graduates, while the Japanese university enrolment rate was reported to be 52·6 % in 2017^(^^46^^)^. Thus, the participants might have had more interest in nutrition or related knowledge compared with the general population^(^^47^^)^. Indeed, compared with dietary intake data of women aged 30–39 years in the National Health and Nutrition Survey in Japan, 2016^(^^31^^)^, which represented the general Japanese population, the fat:carbohydrate energy ratio was slightly lower in the current participants. A previous study has reported that higher nutrition knowledge is associated with lower fat intake^(^^48^^)^. It is possible that the sampling strategy had influenced the relationship (or the lack thereof) between cooking behaviour and nutrient intake. Second, the study sample size was small. We did not perform a power analysis to determine the sample size because the study was designed to examine the influence of the presence of children in the household on the dietary intake of married women, and sample size was thus determined for this purpose^(^^20^^)^. However, according to the power analysis as conducted using G*Power 3^(^^49^^)^, the number of participants required to detect a middle effect size (*f* = 0·25) with a significance level equal to 0·05, a statistical power of 0·8, and with five covariates was estimated to be 128 in total. Third, it is possible that the cooking frequency categories we used are not necessarily the best way to classify the data. To address this concern, we conducted a sensitivity analysis using a different categorisation of cooking frequency and obtained consistent results. Additionally, cooking may have a different meaning for each person^(^^33^^)^ or for people from different cultural backgrounds. In the present study, we examined the frequency of home cooking with dinner as the main meal, similar to previous studies^(^^33^^,^^35^^,^^36^^)^. In contrast, other studies have used both lunch and dinner^(^^4^^)^ or overall frequency^(^^3^^)^ to assess home cooking frequency. Chu *et al*.^(^^12^^)^ have reported that the association between time taken for meal preparation and dietary intakes differed according to meal type (breakfast, lunch, or dinner). The influence of these differences should be addressed in future studies. Ideally, meals other than dinner should be examined in the future to investigate the influence of home cooking behaviour on the quality of dietary intake; we only assessed the habitual dietary intake in the present study. Fourth, we used a questionnaire to assess dietary intake among the participants, which might not be ideal in evaluating nutrient inadequacy, since the questionnaires are limited in their food lists. Finally, our reliance on a cross-sectional dataset does not allow for causal inferences. Confounding factors other than those we examined may have influenced the results. Factors included in this study were based on their primary association between cooking frequency and their effect on dietary intake and the results from previous studies. Further studies are required to look into causal inferences and further determine the role of other factors on nutrient intake.

### Conclusions

This cross-sectional study showed that among Japanese young married women, nutrient intake adequacy did not differ significantly between those who cooked dinner at home daily (daily home cooks) and those who did not (non-daily home cooks). Our findings suggest that in a population with similar demographics a high involvement in cooking the evening meal might not be necessary to achieve adequate nutrient intake. Further studies among various populations and investigating the effects of various barriers to home cooking are required to determine the influence of cooking behaviour on dietary intake.
